# 吉西他滨顺铂联合索拉非尼或安慰剂一线治疗晚期非小细胞肺癌的随机对照研究

**DOI:** 10.3779/j.issn.1009-3419.2011.03.10

**Published:** 2011-03-20

**Authors:** 燕 王, 麟 汪, 雨桃 刘, 舒飞 于, 湘茹 张, 远凯 石, 燕 孙

**Affiliations:** 100021 北京，中国医学科学院北京协和医学院肿瘤医院内科 Department of Medical Oncology, Cancer Institue (Hospital), Chinese Aacademy of Medical Sciences and Peking Union Medical College, Beijing 100021, China

**Keywords:** 肺肿瘤, 化疗, 索拉非尼, 疗效, Lung neoplasms, Chemotherapy, Sorafenib, Response

## Abstract

**背景与目的:**

新药含铂两药联合治疗晚期非小细胞肺癌（non-small cell lung cancer, NSCLC）的疗效已达到平台期，本研究采用前瞻性随机对照的方法比较了吉西他滨顺铂联合索拉非尼或安慰剂一线治疗晚期NSCLC的疗效和安全性。

**方法:**

经细胞学或病理学证实的30例晚期NSCLC患者随机进行吉西他滨顺铂联合索拉非尼或安慰剂治疗，化疗不超过6个周期，有效或稳定的患者继续服用索拉非尼或安慰剂，直至疾病进展或不能耐受不良反应。

**结果:**

实验组（索拉非尼联合化疗组）和对照组（单纯化疗组）的分布基本平衡，两组有效率（response rate, RR）分别为55.6%和41.7%（*P*=0.905）；两组的中位无疾病进展时间（progress-free survival, PFS）相似，分别为5个月和4个月（*P*=0.75）；两组的中位生存时间（overall survival, OS）均为18个月，无统计学差异（*P*=0.68）。不良反应可耐受，实验组的高血压和腹泻发生率较对照组高。Cox多因素回归分析显示，身体状况好（ECOG评分为0分）、肺癌分期早（Ⅲb期）、无肝转移和后续进行酪氨酸激酶抑制剂（tyrasine kinasis inhibitor, TKI）治疗的患者生存明显延长，提示这些因素为良好的预后因素。

**结论:**

在常规吉西他滨顺铂化疗的基础上增加靶向药物索拉非尼并未增加RR、PFS以及OS，后续类似研究需谨慎选择合适的患者。

## Abstract (en)

**Background and objective:**

Platinum-based chemotherapy doublets reached an efficacy plateau in nonsmall- cell lung cancer (NSCLC). This randomized controlled study prospectively assessed the efficacy and safety of cisplatin plus gemcitabine with either Sorafenib or placebo as first-line therapy for NSCLC.

**Methods:**

Thirty patients, which were confirmed advanced NSCLC histologically or cytologically, were randomly assigned to receive up to six cycles of cisplatin plus gemcitabine with sorafenib or placebo. The maintenance of sorafenib or placebo after chemotherapy will continued in patients with response or stable disease until disease progression or unacceptable adverse events.

**Results:**

Overall demographics were balanced between experimental group (sorafenib+chemotherapy) and controlled group (chemotherapy only). Overall response (OS) rate was 55.6% and 41.7% in experimental arm and controlled arm, respectively (*P*=0.905). Median progressivefree survival (PFS) and median overall survial were similar (5 months *vs* 4 months, *P*=0.75; 18 months *vs* 18 months, *P*=0.68). Adverse events were tolerable, though the risk of hypertension and diarrhea was increase in experimental arm. Since patients with ECOG PS 0, stage Ⅲb, no liver metastasis and tyrasine kinasis inhibitor treatment after study had longer survive, these factors seemed to be predictive factors favor of survival in *Cox* regression analyses.

**Conclusion:**

No additional benefit of response rate, PFS or OS were observed from adding targeted agent-sorafenib to regular cisplatin plus gemcitabine chemotherapy. Selecting aproper patients is needed in further study.

目前新药含铂化疗依然是晚期非小细胞肺癌（nonsmall cell lung cancer, NSCLC）的标准一线治疗方案^[1]^，但有效率仅20%-40%，中位生存期为8个月-10个月，其疗效达到了所谓的“平台期”。要想进一步提高有效率和生存期必须寻找与现有作用机制不同的新药物。因此，一系列靶向治疗药物的出现为提高疗效提供了空间，如贝伐单抗、西妥昔单抗等靶向药物与化疗的联合应用已显示出延长生存，改善生活质量的优势。尤其针对表皮生长因子（epidermal growth factor, EGFR）的小分子酪氨酸激酶抑制剂（tyrasine kinasis inhibitor, TKI）一线治疗EGFR基因突变的患者能明显延长无进展生存（progressive-free survival, PFS）^[2]^，但多项大规模Ⅲ期临床研究^[3-6]^却并未显示出此类TKI与化疗联合有更大的生存优势，即便是在EGFR基因突变的人群中也如此。2010年美国ASCO会议发表的一项研究^[7]^结果显示紫杉醇卡铂方案化疗联合厄洛替尼对比单纯TKI一线治疗高选择人群（不吸烟或轻度吸烟的肺腺癌或肺泡细胞癌）疗效相当，联合治疗与单纯厄洛替尼相比未显示生存优势，其中*EGFR*基因突变患者PFS分别为15.7个月和17.2个月，无统计学差异。但对于化疗联合多靶点药物治疗NSCLC能否进一步提高有效率并延长生存是目前研究的热点，尚无定论。

索拉非尼是一种多靶点的TKI^[8]^，能够同时作用于肿瘤细胞的多个分子靶点，包括VEGFR-2、VEGFR-3、PDGFR-β、FLT-3、c-KIT以及C-Raf、B-Raf，可产生多种药理活性，除抑制肿瘤生长外还可以降低微血管密度和面积，明显抑制肿瘤血管生成。前期研究^[9, 10]^表明索拉非尼与细胞毒药物联合治疗晚期NSCLC是有效且安全的。本研究方案采用随机对照的方法比较了索拉非尼或安慰剂与吉西他滨+顺铂联合治疗晚期NSCLC的疗效和安全性。

## 1 资料与方法

### 1.1 入组标准和排除标准

所有患者未经放化疗，均有组织学或细胞学诊断，行为状态按东部肿瘤协作组（Eastern Cooperative Oncology Group, ECOG）评分≤1分，确诊为Ⅲb及Ⅳ期NSCLC，至少有一个可测量病灶，治疗前血红蛋白>9.0 g/dL，中性粒细胞>1, 500/mm^3^，血小板>100, 000/mm^3^，总胆红素≤1.5倍正常值上限，谷丙转氨酶和谷草转氨酶≤2.5倍正常值上限（对于有肝转移的患者≤5倍正常值上限）。均签署知情同意书。已有脑转移、心血管疾病及高血压病不能控制，有出血倾向或凝血功能紊乱表现，最近6个月内出现过血栓形成或栓塞的患者排除在外。

### 1.2 治疗方法

根据ECOG评分和分期随机进行吉西他滨顺铂加索拉非尼或安慰剂治疗。化疗阶段：吉西他滨1, 250 mg/m^2^第1天、第8天静脉注射，顺铂75 mg/m^2^第1天静脉注射，每3周重复，不多于6个周期。同时第1-21天连续口服索拉非尼400 mg每日两次或安慰剂2片每日两次。维持阶段：达到完全缓解、部分缓解和稳定的病例，继续服用索拉非尼或安慰剂，21天为1个周期，直至疾病进展或合并其它疾病而需停止治疗、无法忍耐的毒性、患者拒绝继续服药、以及医生判断可以停止为止。合并用药：顺铂给药前和给药后必须进行水化，化疗中常规止吐治疗，并根据临床需要进行集落刺激因子治疗及其它相应支持治疗。不良反应（adverse events, AEs）按美国国家癌症研究所（National Cancer Institute, NCI）常用毒性标准（Common Terminology Criteria, CTC）评估，血液学毒性CTCAE 4级和非血液学毒性>CTCAE 2级的患者需减量，若仅因血液学毒性需减量，则只减化疗药物剂量；若同时出现血液学和非血液学毒性需减量，则所有药物减量。减量次数不能超过两次，第1次减量至原剂量的80%，第2次减量至原剂量的50%。

### 1.3 疗效评估和毒性评估

采用实体肿瘤疗效评价（Response Evaluation Criteria in Solid Tumors, RECIST）标准，每6周进行影像学肿瘤评估，分为完全缓解（complete response, CR）、部分缓解（partial response, PR）、疾病稳定（stable disease, SD）及疾病进展（progressive disease, PD）。其中CR、PR至少维持4周。疾病控制为CR+PR+SD。所有不良事件均按照NCI CTCAE 3.0版进行分级。生存期（overall survival, OS）为化疗首日起至死亡日或失访日。PFS为化疗首日至影像学证实的进展日或死亡日。

### 1.4 统计学分析

资料采用SPSS 13.0统计软件分析，临床因素与治疗方法的疗效相关分析采用*χ*^2^检验，生存分析包括*Kaplan-Meier*和*COX*多因素回归分析。*P* < 0.05为差异有统计学意义。

## 2 结果

### 2.1 病例特点

中国医学科学院肿瘤医院从2007年6月-2009年1月共筛选39例患者，其中30例患者符合入组条件并完成治疗。筛选失败的主要原因为无症状脑转移（共7例，占总人数的17.9%）。随访截止时间为2010年10月，中位随访时间19个月（范围4个月-40个月）。实验组（化疗+索拉非尼）18例，对照组（化疗+安慰剂）12例（表 1）。实验组的中位化疗周期数为4个周期（范围2个-6个周期），而对照组的中位化疗周期数为5个周期（范围4个-6个周期）。两组在维持阶段的中位周期数均为2个周期（范围0个-12个周期）。期间实验组9例（50.0%）患者进行了1次减量，5例（27.8%）进行了2次减量；对照组进行1次和2次减量的患者分别为7例（58.3%）和3例（25.0%），两组间未出现统计学差异（*P*=0.914）。

**1:** 纳入患者的临床特点 Clinical characteristics of included patients

Characteristic	Gem+Cis+Sorafinib (*n* =18) [*n* (%)]	Gem+Cis+Placebo (*n* =12) [*n* (%)]	*P*
Age (year) [median (range)]	54 (47-62)	56 (37-65)	
Sex			>0.999
Male	10 (55.6)	7 (58.3)	
Female	8 (44.4)	5 (41.7)	
Stage			0.653
Ⅲb	2 (11.1)	1 (8.3)	
Ⅳ	16 (88.9)	11 (91.7)	
ECOG PS			0.694
0	7 (38.9)	3 (25.0)	
1	11 (61.1)	9 (75.0)	
Histology			0.707
Adenocarcinoma	14 (77.8)	10 (83.3)	
Squamous cell carcinoma	3 (16.7)	2 (16.7)	
Large cell carcinoma	1 (5.6)	0	
Smoking history			0.649
Non-smoker	10 (55.6)	8 (66.7)	
Smoker	8 (44.4)	4 (33.3)	
No. of metaststic			0.878
1	9 (50.0)	5 (45.5)	
2	3 (16.7)	5 (45.5)	
3	3 (16.7)	1 (9.1)	
4	1 (5.6)	0	

### 2.2 化疗效果

#### 2.2.1 近期疗效和生存结果

索拉非尼联合化疗组有效率为55.6%，单纯化疗组为41.7%（表 2）；两组的PFS相似，分别为5个月和4个月（图 1）；两组的OS均为18个月，均无统计学差异（图 2）。其中鳞癌患者中接受化疗+索拉非尼治疗的生存时间似乎较单纯化疗延长，中位生存时间（median survival, MS）分别为17个月和7个月，但统计学无差异（*P*=0.620）。实验组和对照组各有2例和1例患者未接受二线治疗，其它27例患者均接受了化疗或TKI二线治疗，其中接受过TKI治疗（只要在后续治疗中接受过TKI治疗的患者，无论是二线治疗或三线以上治疗）的比例分别为实验组61.1%（11例），对照组58.3%（7例）。后续进行过TKI治疗的患者MS为33个月，较从未接受过TKI治疗的患者（17个月）明显延长（*P*=0.014）（图 3）。

**2:** 试验组与对照组间最好疗效评估和生存结果 Comparison of best tumor response and survival between trial group and control group

Response	Gem+Cis+Sorafinib	Gem+Cis+Placebo	*P*
Overall response rate [*n* (%)]	10 (55.6)	5 (41.7)	0.905
Partial response [*n* (%)]	10 (55.6)	5 (41.7)	
Stable disease [*n* (%)]	6 (33.3)	7 (58.3)	
Progressive disease [*n* (%)]	2 (11.1)	0 (0)	
Median PFS (month)	5	4	0.750
MS for All (month)	18	18	0.680
MS for squamous (month)	17	7	0.620
PFS: progressive-free survival; MS: median survival.			

**1:**
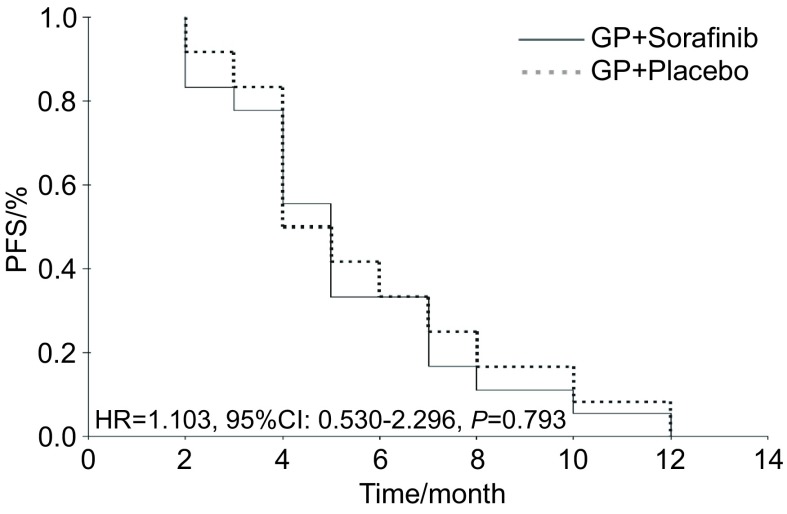
所有患者PFS的*Kaplan-Meier*曲线 *Kaplan-Meier* curve of PFS for overall patients

**2:**
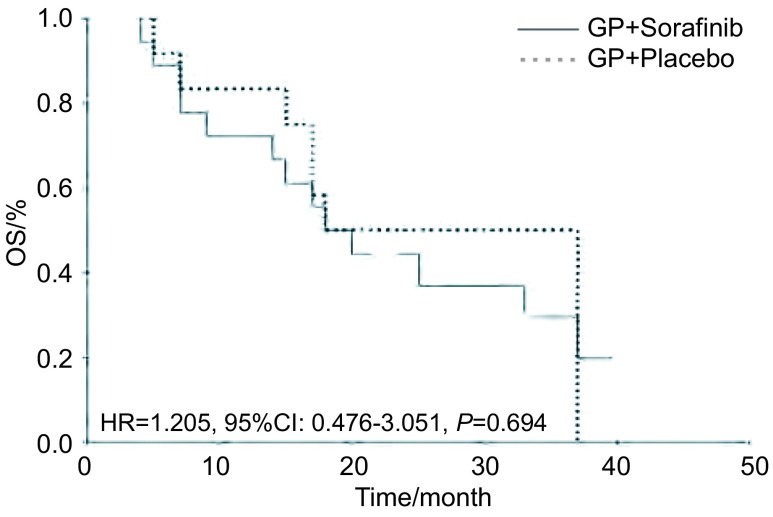
所有患者总生存期的*Kaplan-Meier*曲线 *Kaplan-Meier* curve of overall survival (OS) for overall patients

**3:**
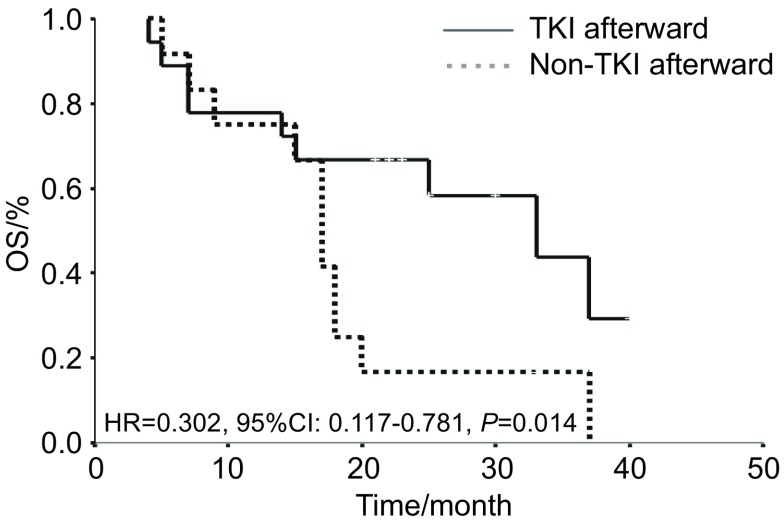
用过或未用过TKI的患者OS的*Kaplan-Meier*曲线 *Kaplan-Meier* curve of OS for patients with or without TKI treatment

#### 2.2.2 不良反应

本组病例中没有出现因为不能耐受不良反应而出组的情况。主要的血液学毒性包括中性粒细胞减少、血小板减少及贫血。两组发生CTCAE 3、4级血液学毒性的比例无明显差异（分别为72.2%和66.7%，*P*>0.999）。主要的非血液学毒性包括胃肠道不良反应、乏力、脱发等，多数为CTCAE 1级-2级，其中实验组腹泻和高血压的发生率明显高于对照组（表 3）。实验组CTCAE 3级-4级非血液学毒性似乎高于对照组，但无统计学差异（66.7% vs 25.0%, *P*=0.060）。其它如胆红素升高、低磷血症和耳鸣等症状在对照组中未出现，但两组的差异未达到统计学意义。其中仅实验组出现1例腺癌患者致死性肺栓塞，不能排除与索拉非尼有关。

**3:** 试验组与对照组不良反应比较 Comparison of adverse events between trial group and control group

Adverse event	Gem+Cis+Sorafinib (*n* =18)				Gem+Cis+Placebo (*n* =12)			*P*
	All grade[*n* (%)]	Grade 3[*n* (%)]	Grade 4[*n* (%)]		All grade[*n* (%)]	Grade 3[*n* (%)]	Grade 4[*n* (%)]	
Hematologic toxicities								
Leukopenia	16 (88.9)	8 (44.4)	2 (11.1)		12 (100.0)	6 (50.0)	2 (16.7)	0.589
Thrombocytopenia	16 (88.9)	5 (27.8)	4 (22.2)		6 (50.0)	1 (8.3)	1 (8.3)	0.295
Anemia	13 (72.2)	3 (16.7)	2 (11.1)		9 (75)	2 (16.7)	1 (8.3)	0.638
Nonhematologic toxicties								
Nausea/Vomiting	18 (100.0)	7 (38.9)	0 (0)		12 (100.0)	3 (25.0)	0 (0)	0.287
Diarrhea	18 (100.0)	88 (44.4)	1 (5.6)		1 (8.3)	0 (0)	0 (0)	0.023
Fatigue	16 (88.9)	0 (0)	0 (0)		3 (25.0)	0 (0)	0 (0)	0.451
Anorexia	18 (100.0)	8 (44.4)	0 (0)		3 (25.0)	3 (25.0)	0 (0)	0.282
Alopcia	18 (100.0)	0 (0)	0 (0)		12 (100.0)	0 (0)	0 (0)	0.503
Hypertension	7 (38.9)	1 (5.6)	0 (0)		0 (0)	0 (0)	0 (0)	0.024
Rash/desquamation	3 (16.7)	1 (5.6)	0 (0)		2 (16.7)	0 (0)	0 (0)	0.528
Bilirubinemia	3 (16.7)	0 (0)	0 (0)		0 (0)	0 (0)	0 (0)	0.250
Hypophosphatemia	2 (11.1)	1 (5.6)	0 (0)		0 (0)	0 (0)	0 (0)	0.490
Tinnitus	3 (16.7)	0 (0)	0 (0)		0 (0)	0 (0)	0 (0)	0.329

#### 2.2.3 OS预后因素分析

对全部30例患者性别（男*vs*女）、吸烟状况（吸烟*vs*不吸烟）、ECOG评分（1 *vs* 0）、病理类型（鳞癌*vs*非鳞癌）、肺癌分期（Ⅲb *vs* Ⅳ）、肝转移（有*vs*无）、治疗效果、后续TKI治疗（是*vs*否）等多个可能影响预后的因素先进行单因素分析，再进行*COX*多因素回归分析，结果显示ECOG评分为0分、肺癌分期为Ⅲb、无肝转移和后续进行了TKI治疗为影响预后的良好独立因素（表 4）。

**4:** *COX*回归分析中的预后因素 Predictive factors of survival in *Cox* Regression analysis

	B	SE	Wald	df	Sig.	Exp(B)	95%CI for Exp(B)	
							Lower	Upper
Stage (Ⅲb *vs* Ⅳ)	-2.149	0.713	9.069	1	0.003	0.117	0.029	0.472
ECOG (1 *vs* 0)	1.360	0.634	4.597	1	0.032	3.897	1.124	13.512
Liver metastasis (yes *vs* no)	2.540	0.843	9.083	1	0.003	12.682	2.431	66.160
TKI (yes *vs* no)	-1.197	0.485	6.098	1	0.014	0.302	0.117	0.781
< 1 means favor to the factor forward，>1 means favor to the factor afterward; TKI: tyrasine kinasis inhibitor.								

## 3 讨论

本组研究结果说明，在标准的吉西他滨顺铂化疗方案基础上联合多靶点药物索拉非尼一线治疗晚期NSCLC并未提高患者的有效率和生存。与单纯化疗相比，虽然实验组的有效率从数值上较高（分别为55.6%和41.7%），但统计学并无差异（*P*=0.905）。但从维持阶段的治疗来看，实验组中位治疗周期数仅2个周期，说明疗效持续时间不长，病变很快进展，从而导致PFS也未能延长（中位PFS分别为5个月和4个月，*P*=0.750）。此外，由于两组中的大多数患者（分别为61.1%和58.3%）在以后的治疗中均进行过TKI治疗，因此两组的OS均长达18个月，高于目前循证医学提供的最好生存数据（13个月）。

本组研究数据与Scagliotti^[11]^今年报道的一项大规模随机Ⅲ期临床试验结果非常相似。该试验因中期分析结果显示索拉非尼组无生存优势而提前终止。共926例未接受过化疗的NSCLC患者随机接受了紫杉醇卡铂联合或不联合索拉非尼治疗的疗效，对于治疗有效的患者继续使用索拉非尼治疗直至疾病进展。联合索拉非尼治疗组与单纯化疗组相比，无进展生存时间分别为5.1个月和5.4个月（*P*=0.433）；总生存时间分别为10.7个月和10.6个月（*P*=0.915）。该研究在亚组分析中发现，与单纯化疗相比，进行化疗+索拉非尼联合治疗的鳞癌患者不良反应尤其是致死性出血的发生率较高而PFS和OS均较短。但本研究无类似发现，可能是因为病例数太少的缘故。

目前除贝伐单抗联合紫杉醇或卡铂可以改善特定（非鳞癌、无脑转移以及无出血）的晚期NSCLC患者的生存以外^[12]^，在前期研究^[13, 14]^中显示出其它多种与化疗联合有较好疗效的靶向药物，均未能在以后的大规模随机临床研究中重复同样的结果，即：与单用化疗的一线治疗相比，联合治疗并无生存获益。推测原因，一是靶向药物和化疗药物影响不同的细胞周期，反而产生拮抗作用或增加不良反应；二是不同的化疗方案与靶向药物的协同性不一样。比如同样是贝伐单抗，当与紫杉醇卡铂联合时可以延长生存，而与吉西他滨顺铂联合时却无生存受益^[15]^；三是可能未根据药物针对的靶点预选合适的患者，即在非特定人群中进行靶向治疗，因此难以发挥应有的疗效，但遗憾的是，目前索拉非尼还没有明确的生物标记物。根据生物标志物决定靶向药物的BATTLE研究^[16]^中，索拉非尼治疗*K-ras*突变的患者取得了较好疗效，提示寻找有效靶点是靶向治疗最为关键的步骤。

耐药是单靶点TKI广泛应用后引起关注的重要问题。目前认为除了继发T790M突变是导致继发性耐药的原因之外，*C-met*突变是另一耐药的主要原因。多靶点TKI如索拉非尼、舒尼替尼等可作用于包括met在内的多个靶点，因此有望克服这一难题。2010欧洲肿瘤内科年会一项研究^[17]^报道，舒尼替尼与厄洛替尼联合应用于一线治疗失败的晚期NSCLC，有效率及PFS均明显优于单用厄洛替尼。此外，另一项研究^[18]^结果显示met受体拮抗剂与厄洛替尼联合可使*met*基因突变的患者发生疾病进展的风险下降44%。

值得注意的是，本研究中无论是实验组还是对照组，分别有77.8%和83.3%的患者进行了减量，其中近四分之一的患者（分别为27.8%和25.0%）进行了2次减量。血液学毒性均高达70%以上，其中3度-4度中性粒细胞减少的发生率分别为55.5%和66.7%，3度-4度血小板减少分别为50%和16.6%，3度-4度贫血分别为27.8%和25%；非血液学毒性中胃肠道反应较明显，两组相似，3度-4度恶心呕吐的发生率分别为38.9%和25%。虽然经过减量和对症支持治疗，未出现因不良反应而停止治疗的情况，但以上数据说明在临床应用中制定吉西他滨1, 250 mg/m^2^的剂量时需格外谨慎，必须密切观察和处理不良反应。但剂量高也许是本研究中有效率高于既往研究的原因之一。此外，实验组乏力、高血压、低磷血症、耳鸣的发生率较单纯化疗组高，可能与索拉非尼有关。

总之，虽然本研究为阴性结果，但由于存在一些不足之处，如例数较少，亦无法进行亚组分析，因此得出的结论说服力有限。此外，索拉非尼单药在二线以上的晚期NSCLC治疗中确实表现出一定疗效，以后能否应用于一线治疗尚有待进一步研究。值得注意的是需要谨慎选择合适的患者。
